# Strength and Brittleness of Interfaces in Fe-Al Superalloy Nanocomposites under Multiaxial Loading: An ab initio and Atomistic Study

**DOI:** 10.3390/nano8110873

**Published:** 2018-10-24

**Authors:** Petr Šesták, Martin Friák, David Holec, Monika Všianská, Mojmír Šob

**Affiliations:** 1 Institute of Physics of Materials, Academy of Sciences of the Czech Republic, Žižkova 22, CZ-616 62 Brno, Czech Republic; sestak@fme.vutbr.cz (P.Š.); vsianska@ipm.cz (M.V.); mojmir@ipm.cz (M.S.); 2 Central European Institute of Technology, CEITEC BUT, Brno University of Technology, Purkyňova 123, CZ-612 00 Brno, Czech Republic; 3 Department of Physical Metallurgy and Materials Testing, Montanuniversität Leoben, Franz-Josef-Strasse 18, A-8700 Leoben, Austria; david.holec@unileoben.ac.at; 4 Central European Institute of Technology, CEITEC MU, Masaryk University, Kamenice 5, CZ-625 00 Brno, Czech Republic; 5 Department of Chemistry, Faculty of Science, Masaryk University, Kotlářská 2, CZ-611 37 Brno, Czech Republic

**Keywords:** Fe-Al, superalloys, order, tensile strength, elasticity, ab initio, stability, nanocomposite

## Abstract

We present an ab initio and atomistic study of the stress-strain response and elastic stability of the ordered Fe${}_{3}$Al compound with the D0${}_{3}$ structure and a disordered Fe-Al solid solution with 18.75 at.% Al as well as of a nanocomposite consisting of an equal molar amount of both phases under uniaxial loading along the [001] direction. The tensile tests were performed under complex conditions including the effect of the lateral stress on the tensile strength and temperature effect. By comparing the behavior of individual phases with that of the nanocomposite we find that the disordered Fe-Al phase represents the weakest point of the studied nanocomposite in terms of tensile loading. The cleavage plane of the whole nanocomposite is identical to that identified when loading is applied solely to the disordered Fe-Al phase. It also turns out that the mechanical stability is strongly affected by softening of elastic constants $C^{\prime }$ and/or $C_{66}$ and by corresponding elastic instabilities. Interestingly, we found that uniaxial straining of the ordered Fe${}_{3}$Al with the D0${}_{3}$ structure leads almost to hydrostatic loading. Furthermore, increasing lateral stress linearly increases the tensile strength. This was also confirmed by molecular dynamics simulations employing Embedded Atom Method (EAM) potential. The molecular dynamics simulations also revealed that the thermal vibrations significantly decrease the tensile strength.

## 1. Introduction

Iron-aluminium-based materials containing either Fe${}_{3}$Al and/or FeAl intermetallic compounds represent one of the most promising classes of metallic alloys intended for high-temperature structural applications. They are known for many excellent properties, e.g., (i) resistance to oxidation [1] or with respect to various molten salts [2]; (ii) relatively low density; (iii) electrical resistivity and (iv) low cost of raw materials [3,4,5]. On the other hand, their wider use is hindered by their low ductility at ambient temperatures and a drop of the strength at elevated temperatures [5]. Regarding the former, it has been shown that it is caused by an extrinsic effect, in particular hydrogen atoms produced by the reaction of water vapor with aluminum at the surface of the specimen [6,7]. If it is not for this environmental embrittlement, Fe${}_{3}$Al is seen in experiments to have decent ductility [8,9]. Recently, there is a renewed interest in Fe-Al-based materials containing higher number of chemical species and/or phases [1,10,11,12,13,14,15,16,17,18,19].

A special sub-class of Fe-Al-based nanocomposites [20,21,22,23,24,25,26,27,28,29,30,31,32,33] are those consisting of two phases, ordered Fe${}_{3}$Al with the D0${}_{3}$ structure and a disordered Fe-Al solid solution with about 18.75 at.% Al (see, e.g., Refs. [22,24,34]). These phases co-exist in the concentration range from about 19 to about 25 at.% of Al (see the original Fe-Al phase digram by Kattner and Burton [35]) reproduced, for example, in an excellent review by Sundman and co-workers [36]. Importantly, the transformation of phases in the Fe-Al system is particularly complicated and the final state of samples is very sensitive to many factors including thermo-mechanical history [37]. The co-existence of Fe${}_{3}$Al compound and a disordered Fe-Al solid solution is best experimentally confirmed by transition electron microscopy (TEM) technique which is sensitive to anti-phase boundaries (APBs) as these have a specific character in Fe${}_{3}$Al and a different one in other, at least partly ordered, Fe-Al phases. In particular, Oguma et al. [38] developed a time-dependent Ginzburg-Landau (TDGL) formulation for the ordering processes of B2 and D0${}_{3}$ types in binary alloy systems. Specifically in the case of Fe-Al, numerical simulations of the kinetic equations have been performed for concurrent ordering and phase separation to disordered A2 and ordered D0${}_{3}$ and helped to explain TEM observations. This combined theoretical and experimental analysis identified round/oval droplets of the disordered Fe-Al phase formed on the expense of diminishing amount of ordered Fe${}_{3}$Al phase. The rounded shape of these droplets indicate that the interface energy is not sensitive to crystallographic orientation and, therefore, the (001) interfaces studied in this paper are equally probable as others (when mechanical properties can admittedly be orientation-sensitive).

**Figure 1:**
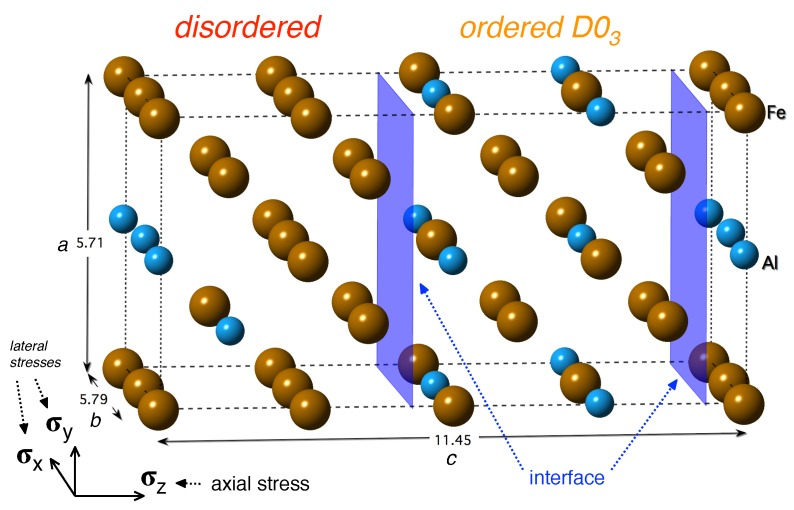
A schematic visualization of a supercell used in our ab initio calculations. The 32-atom supercell contains a disordered Fe-Al phase (left-hand side) and an ordered Fe${}_{3}$Al compound with the D0${}_{3}$ structure (right-hand side). The interface between both phases is highlighted by the blue planes.

Papers related to first-principles calculations of these two-phase coherent nanocomposites are rather rare as most of the previous studies focused on individual phases appearing in the Fe-Al phase diagram (see a few selected examples listed below). In order to fill in this gap, we study in this work thermodynamic, structural and elastic properties of interfaces between these two phases (see Figure 1) without any external load as well as under extreme uniaxial loading conditions leading to the instability of these composites. In particular, we compare the properties of the two-phase nanocomposite with properties of both constituting phases.

Let us summarize the main results of the previous studies on these constituting phases first. Watson and Weinert [39] reported heats of formation for binary and ternary 3d transition-metal (Ti, V, Fe, and Ni) aluminides using the local density approximation (LDA). They found their predictions in the case of Fe aluminides overestimated by about 0.15 eV/atom when compared with experimental data. As the most likely reason they identified a poor description of bcc Fe by LDA (a fact which lead us to use the generalized gradient approximation (GGA) in our work). Another calculations by Gonzales-Ormeno et al. [40] were performed employing Perdew-Burke-Ernzerhof (PBE) parametrization [41] of GGA and Full Potential - Linear Augmented Plane Wave method (FP-LAPW). The computed formation energies of the D0${}_{3}$ (Fe${}_{3}$Al) and B2 (FeAl) compounds show excellent agreement with available calorimetric data on standard enthalpies of formation of Fe-Al alloys up to 50 at.% aluminium.

Lechermann et al. [42] demonstrated that neither LDA nor PBE parametrization of GGA can correctly reproduce the D0${}_{3}$ structure as the ground state of Fe${}_{3}$Al and both above mentioned parametrizations of the exchange-correlation energy prefer the L1${}_{2}$ structure (at T = 0 K and in the case of defect-free static lattices without any collective excitations). Subsequently, in another paper [43], Lechermann and co-workers studied electronic correlations and magnetism in Fe${}_{3}$Al employing local density approximation with an additional energy term (LDA+U) and correctly obtained the D0${}_{3}$ structure as the ground-state structure of Fe${}_{3}$Al. Similarly, Connetable and Maugis [44] calculated structural, magnetic, elastic and vibrational properties of Fe${}_{3}$Al employing PBE parametrization [41] of the GGA and found out that Fe${}_{3}$Al has a lower energy in the L1${}_{2}$ structure than in the experimentally observed D0${}_{3}$ structure.

As far as solid solutions of Al and Fe are concerned, Amara and co-workers [45] performed first-principles calculations to study the electronic structure and energetics of the dissolution of aluminum in α-iron and the interaction between Al atoms and vacancies. It was found that the stability of these complexes is mainly driven by strong Al-vacancy attractions whereas Al-Al interactions are repulsive. Liu et al. [46] calculated the difference in vibrational entropy between chemically disordered and ordered Fe-Al compounds. Kulikov et al. [47] have studied the electronic structure of disordered bcc Fe${}_{x}$Al${}_{1-x}$ (0.4 <*x*< 0.75) alloys around the equiatomic stoichiometry, as well as of the ordered B2-structure FeAl phases with point defects employing the coherent potential approximation within the Korringa-Kohn-Rostoker (KKR) method for the disordered case and the tight-binding linear muffin-tin orbital (TB-LMTO) method for the intermetallic compounds. Studying in particular the onset of magnetism in Fe-Al they found the appearance of large local magnetic moments associated with the transition metal antisite defect in FeAl, in agreement with the experimental findings.

Furthermore, Friák and Neugebauer [48] performed an ab initio study of a dense set of Fe-Al compositions and local atomic arrangements in order to explain the anomalous volume-composition dependence in Fe-Al alloys. They found that the spin-polarized calculations for Fe-rich compounds reproduce very well the anomalous lattice-constant behavior in contrast to both the nonmagnetic and fixed-spin-moment calculations that result in nearly linear trends without any anomaly. The change in magnetism of iron atoms caused by an increasing number of Al atoms in the first coordination spheres was thus identified as the decisive driving force of the anomalous behavior.

Regarding other published papers, Fähnle et al. [49] applied cluster-expansion method to predict the phase diagram for the system Ni-Fe-Al, Friák et al. [50] studied an impact of solutes (in particular Ti additions) on the elastic properties of Fe${}_{3}$Al both theoretically by first-principles calculations and experimentally by ultrasonic measurements, Kirklin et al. [51] performed a high-throughput computational search for strengthening precipitates in alloys including Fe matrix and Fe-Al-based compounds, Airiskallio et al. [52] studied corrosion resistance of Fe–Al and Fe-Al-Cr alloys in oxidizing environment using the Exact Muffin-Tin Orbitals (EMTO) method as an alternative of screened Korringa-Kohn-Rostoker (KKR) method, Medvedeva et al. [53] calculated impact of Al and C on the stacking fault energies in fcc Fe using generalized gradient approximation and the projector-augmented waves (PAW) potentials [54] implemented in the same code VASP [55] as we use (see below), Čížek et al. [56] used quantum-mechanical calculations when characterizing quenched-in vacancies in Fe-Al alloys, Ipser et al. [57] developed a statistical-thermodynamic model for intermetallic phases with D0${}_{3}$-structure and Kellou et al. [58] used DFT-GGA calculations to study the magnetic properties Fe${}_{3}$Al and Fe${}_{3}$AlX (X = H, B, C, N, O) compounds.

As an evidence of how intensive have been first principles calculations applied in the case of Fe-Al-based materials it should be noted that the papers listed above represent only minor part of all publications focused on individual phases within the Fe-Al binary system so far.

This paper is organized as follows. After the Introduction, Section 2 describes computational details. The results obtained are discussed in Section 3 and Section 4 then presents the conclusions and summarizes the whole paper.

## 2. Materials and Methods

The present simulations were performed with the help of ab initio total-energy and molecular-dynamics program VASP (Vienna ab initio simulation package) developed at the Fakultät für Physik, Universität Wien [55]. In the presented study the electron interactions were described with the projector-augmented waves (PAW) potentials as supplied with the VASP code [54] and the exchange correlation energy was evaluated by means of the generalized gradient approximation (GGA) with parametrization of Perdew-Wang [59]. A Methfessel-Paxton method of the first order was adopted with a smearing width of 0.1 eV. Importantly, our setting prefers the experimentally observed D0${}_{3}$ structure of Fe${}_{3}$Al over the L1${}_{2}$ structure by 5 meV/atom.

The sampling of the Brillouin zone was done using Monkhorst-Pack [60] grids 5 × 5 × 1, 5 × 5 × 3 and 5 × 5 × 5 for the simulation cells containing 64 (double cell - composite), 32 (composite) and 16 (D0${}_{3}$ and disordered phases) atoms, respectively. The convergence steps in the DFT cycle were considered as self-consistent when the differences in energy between two consequent steps was below $10^{-6}$ eV/(sim. cell) and the plane wave basis set was expanded with the cut off energy 350 eV. During the simulations it was necessary to optimize all atomic positions and the cell shape. The atomic positions were optimized using the internal VASP procedure until all forces between atoms were lower than 10 meV/Å while for the optimization of the cell shape we used our own external program that cooperated with the VASP code via reading its output files and writing new structure input files. In stress control calculations this program allowed us to relax the stress tensor components to their targeted values within the selected tolerance. In this work this tolerance was set to be ±0.10 GPa. In all present calculations, magnetism was included via spin polarization and all simulations always started in ferromagnetic state.

The simulation supercell used in the present work is depicted in Figure 1 together with the cell dimensions and orientation of the coordinate system. This cell is assembled from two parts where the first one corresponds to the ordered Fe${}_{3}$Al phase with the D0${}_{3}$ structure and the second one to the disordered Fe-Al, i.e., a solid solution of Al atoms in bcc Fe with 18.75 at.% Al and the atoms distributed according to the special quasi-random structure (SQS) concept developed by Zunger et al. [61]. The SQS concept is based on the idea that atoms are distributed in a rather small periodically repeated supercell in such a way that their statistical characteristics (average occupations of nearest-neighbor shells, so-called Warren-Cowley short-range order (SRO) parameters) mimic those in an ideal disordered solid solution of atoms with the same chemical composition in an infinitely large system. In order to achieve this goal up to, e.g., second, third or fourth coordination shell, different local atomic environments are typically included. For example, the SQS-part of Figure 1 contains Al atoms distributed in way that they mutually form the first and second nearest neighbor pairs. This is in a clear contrast to the Fe${}_{3}$Al-part of Figure 1 which contains Al atoms forming solely the third nearest neighbor pairs (a characteristic feature of the D0${}_{3}$ structure). The interface between both parts (the blue plane) is located in the middle and due to application of the periodic boundary conditions in ab initio simulations is also located at the cell edges with respect to the *z* direction.

The strength characteristics in this work are represented by the stress-strain responses obtained from the tensile loading that was always oriented perpendicular to the interface (or along the equivalent directions for the perfect crystals). The stress and strain acting perpendicular to the interface are denoted as the axial stress $\sigma_{\mathrm{ax}}$ and axial strain $\varepsilon_{\mathrm{ax}}$, respectively. The maxima at the stress-strain dependence can be considered as the tensile strength $\sigma_{\mathrm{ts}}$. For comparison, the tensile strength characteristics were not only determined for the simulation cell depicted in Figure 1 but also for the perfect D0${}_{3}$ and the disordered phases. It must be pointed out that we have simulated behavior of defect-free systems while some crystal defects or instabilities might lower the tensile strength (e.g., dislocations, grain boundaries, phonon or elastic instability, etc.) before reaching the maxima at the stress-strain curve. However, quantum-mechanical phonon calculations, which can assess temperature effect, are computationally very demanding and, therefore, we leave it for future studies.

To obtain at least partial information related to finite-temperature properties, we complement our ab initio calculations with the molecular dynamics simulations (MD) to see if and how temperature decrease the tensile strength $\sigma_{\mathrm{ts}}$. For the MD simulations, we used the code LAMMPS (Large-scale Atomic/Molecular Massively Parallel Simulator) [62] with the embedded-atom method (EAM) potential type [63].

To check the precision of the MD potential we performed the comparison of the stress-strain dependencies and the related tensile strengths with those obtained from first-principles calculations. This cross-checking of atomistic and quantum-mechanical methods was realized under quasi-static MD simulations which means that the atomic motions were set to absolute zero (no kinetic energy). All the atoms in the simulation were thus kept frozen and the tensile tests were realized in a similar way as in the case of ab initio simulations, e.g., via homogeneous dilatation of the simulation cell followed by the optimization of all atomic positions and the cell shape at each strain increment. The Polak-Ribiere version of the conjugate gradient (CG) algorithm was used for the atomic optimization during the quasi static simulations performed by LAMMPS. For the MD simulations (non quasi-static) we set the time step to 2 fs and the strain rate was chosen 10${}^{-4}$/ps.

In quantum-mechanical calculations, the stress values can be computed from the total energy changes between two strain increments according the following formula

(1) $$\sigma_{\mathrm{ax}}=\frac{1}{V}\frac{dE_{\mathrm{tot}}}{d\varepsilon_{\mathrm{ax}}}$$

where $dE_{\mathrm{tot}}$ is the total energy change between two consequent deformation steps, dε is the increment of the axial strain and *V* corresponds to the volume of the simulation cell (a recent review of strength studies may be found in Ref. [64]). Another way how to obtain the axial stress is to read its value directly from the VASP or LAMMPS outputs. The ab initio results presented in this work are based on the stress tensor that was obtained directly from the VASP output (OUTCAR). This is due to fact that we read not only the axial stress but also all remaining stress tensor components which cannot be obtained from Equation (1). Because the axial stress computed from the total energy changes is less sensitive to the settings of first-principles simulations we compare the values from both approaches to check whether the simulation settings are sufficient to obtain reliable results.

The deformation of the supercell in the quantum-mechanical and MD (quasi-static) simulations was realized via homogeneous straining along the axis perpendicular to the interface. During each strain increment the cell shape and ionic positions were optimized within the cell according to selected deformation model (for a detailed review, see e.g., Refs. [65,66]). Because the real crystal structures are mostly subjected to complex loading conditions, we systematically studied the mechanical responses under optimized uniaxial deformation (OUD), optimized uniaxial loading (OUL) and also under superimposed lateral stress $\sigma_{\mathrm{lt}}$.

The OUD mode is based on the strain increments along only one direction while the dimensions of the crystal along other two directions remain constant during the entire tensile test. Thus, the simulation cell changes its shape only in one direction ($\varepsilon_{x}=0,\varepsilon_{y}=0,\varepsilon_{\mathrm{ax}}\neq 0$). Here, we would like to point out, that the OUD model usually leads to the triaxial loading state [65,66]. On the other hand, the OUL model comprises relaxation of the lateral stresses and therefore all three dimensions change during the deformation ($\varepsilon_{x}\neq 0,\varepsilon_{y}\neq 0,\varepsilon_{ax}\neq 0$). The last deformation model is very similar to the OUL model and difference is that both lateral stresses are not relaxed close to zero value. Instead of that, they are relaxed close to predefined certain values which are kept constant for the entire tensile test. Because both lateral stresses are chosen to be equal in all our models ($\sigma_{x}$ = $\sigma_{y}$) we marked them as lateral stress $\sigma_{\mathrm{lt}}$. In this work, the lateral stresses were chosen from a range starting from 0 GPa to 20 GPa with a step 5 GPa. Due the cubic crystal symmetry and the loading conditions in the [001] direction there are no shear stresses and therefore the stress tensor acquires a following simple form:

(2) $$\hat{\sigma }=\left|\begin{array}{ccc} \sigma_{\mathrm{lt}} & 0 & 0\\ 0 & \sigma_{\mathrm{lt}} & 0\\ 0 & 0 & \sigma_{\mathrm{ax}} \end{array}\right|$$

## 3. Results

### 3.1. Mechanical Properties of Individual Phases (D0${}_{3}$ and Disordered)

**Figure 2:**
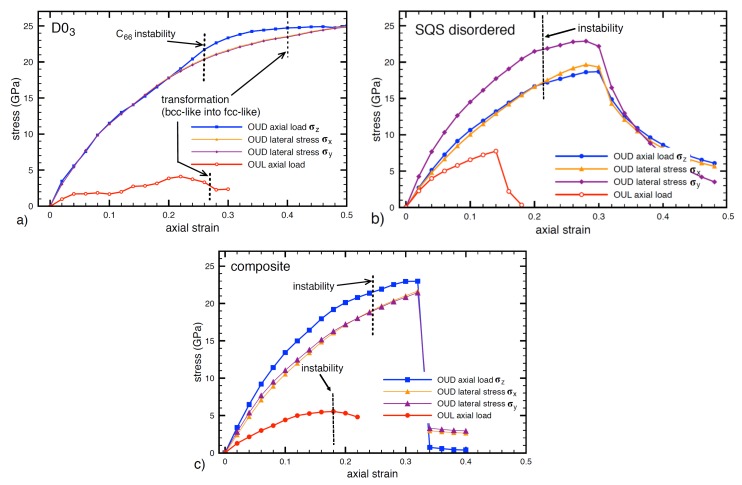
The stress-strain dependencies obtained from ab initio simulations for the uniaxial deformation (OUD) and uniaxial loading (OUL) deformation models for perfect Fe${}_{3}$Al with the D0${}_{3}$ structure (**a**), a disordered Fe-Al phase (**b**) and their nanocomposite (**c**). The blue, magenta and orange curves represent axial and two transverse stresses for the OUD model while the red one belongs to the axial load of the OUL model. Elastic instabilities are marked by black dashed lines.

As the first step, we obtained the stress-strain characteristics of the D0${}_{3}$ and the disordered phases subjected to the uniaxial deformation (OUD) and the uniaxial loading (OUL) deformation modes. The resulting stress-strain responses for the D0${}_{3}$ phase are depicted in Figure 2a. Surprisingly, all three curves for the OUD model (the axial ($\sigma_{\mathrm{ax}}$) and two laterals ($\sigma_{x}$ and $\sigma_{y}$)) are almost identical with respect to the axial strain $\varepsilon_{\mathrm{ax}}$ values except the location close to maxima where the axial stress $\sigma_{\mathrm{ax}}$ has a slightly higher values than the lateral ones.

The fact that the uniaxial deformation (OUD) leads to nearly identical values of all three normal stresses, i.e., $\sigma_{x}=\sigma_{y}=\sigma_{\mathrm{ax}}$, means that the loading is almost hydrostatic (the stresses are nearly equal). We would like to point out that the material response to the uniaxial deformation is usually represented by smaller values of the lateral stresses with respect to the axial one (see, e.g., results for perfect Ni crystal in direction [001] and [1$\bar{2}$0] in Ref. [65]).

It is worth noting that the expected maxima of the axial strain for the OUD model in Figure 2 is located in rather large values of the axial strain. The strain in the direction [001] is, in fact, comparable with deformations related to the Bain’s transformation path when the bcc lattice transforms into the fcc one. Indeed, in the case of bcc-based D0${}_{3}$ structure these conditions occur for the OUD model when the axial strain reaches value ($\sqrt{2}-1$) [67,68,69]. Beyond this value the structure cannot be considered as D0${}_{3}$ and hence the axial stress at this transformation point may be considered as the theoretical tensile stress $\sigma_{\mathrm{ts}}$. The axial strains corresponding to the Bain’s deformation path are marked in Figure 2a by black vertical dashed line and the corresponding tensile strength $\sigma_{\mathrm{ts}}^{\mathrm{OUD}}$ for the OUD model is determined in the case of Fe${}_{3}$Al to be 24.7 GPa. There is also a question whether the D0${}_{3}$ is elastically stable for such large strain values. We will focus on this question in Section 3.5.

Regarding the uniaxial loading (OUL), it revealed a completely different response (see Figure 2a). As a result of the optimization of the lateral stresses to zero values during the entire deformation path, e.g., allowing the Poisson’s contraction, OUL predicts remarkably lower values of the axial stress $\sigma_{\mathrm{ax}}$ and the related tensile strength ($\sigma_{\mathrm{ts}}^{\mathrm{OUL}}$ = 4.1 GPa), i.e., only 17 % of the value found in the case of the OUD model. As far as the strains corresponding to the bcc-to-fcc transition according to the Bain’s deformation path are concerned, they are marked in Figure 2a by black vertical dashed line but the value for the OUL model does not have any impact on the tensile strength due to its location after the maximum of the stress.

Importantly, from the comparison of the OUD and OUL approaches above it is evident, that the strength $\sigma_{\mathrm{ts}}$ of the D0${}_{3}$ phase is very sensitive to the lateral stresses $\sigma_{\mathrm{lt}}$ and even small increases/decreases of these stresses increase/decrease the tensile strength $\sigma_{\mathrm{ts}}$. For this reason, we investigated the influence of $\sigma_{\mathrm{lt}}$ on the tensile strength $\sigma_{\mathrm{ts}}$ in more detail and these results are described and discussed in Section 3.3. The stress-strain dependence for the OUL model also shows a plateau for the strain values in the range 0.05–0.1. This plateau means zero value of the corresponding elastic constant in this region and possibility of some structure instability. Hence, it is a question which maximum at the stress-strain should be considered as the theoretical tensile strength $\sigma_{\mathrm{ts}}$.

To answer this question we computed elastic constants for several points of the stress-strain curves and also employed the molecular dynamics simulations to include temperature effect (lattice vibration) into the simulations. These results are discussed in Section 3.4 and Section 3.5. Because the stress-strain curve in Figure 2a for the OUL model contains the plateau and has a different shape compared to the other structures there is a question whether the precision of the ab initio simulations in this case is sufficient. For this reason, we also performed the tensile test using very precise simulation settings (the cut-off energy was increased to 500 eV, the tolerance of the DFT cycle to 10${}^{-8}$ eV and the k-point grid to 11 × 11 × 11); the obtained stress-strain curve is practically identical to that one obtained earlier.

In the next stage, we performed the tensile tests for the disordered Fe-Al phase with 18.75 at.% Al and the obtained stress-strain behavior is illustrated in Figure 2b. For the disordered phase and the OUD model the tensile strength $\sigma_{\mathrm{ts}}^{\mathrm{OUD}}$ was determined to be equal to 22.9 GPa which is slightly lower than for the D0${}_{3}$ (24.7 GPa). On the contrary, the tensile strength for the OUL reach almost doubled value ($\sigma_{\mathrm{ts}}^{\mathrm{OUL}}$ = 7.8 GPa) when compared with the Fe${}_{3}$Al phase ($\sigma_{\mathrm{ts}}^{\mathrm{OUL}}$ = 4.1 GPa). Thus, the disordered phase is less sensitive to the transversal stresses than the ordered Fe${}_{3}$Al phase and it has higher tensile strength $\sigma_{\mathrm{ts}}^{\mathrm{OUL}}$ under the uniaxial loading. We note that the lateral stresses $\sigma_{x}$ and $\sigma_{y}$ reach very high values close to the axial one ($\sigma_{\mathrm{ax}}$). This indicates that the uniaxial deformation also leads to a stress state which is very close to the hydrostatic one (as mentioned above for the Fe${}_{3}$Al phase). Also, the stress-strain curve for the OUL is smooth, does not contain any irregularities and its maximum point is followed by sudden drop which indicates a fracture in the structure. The obtained tensile strengths $\sigma_{\mathrm{ts}}$ are summarized in Table 1 for all Fe-Al alloys and the deformation models applied in this work. The OUD value of the tensile strength of the Fe${}_{3}$Al compound in the [001] direction $\sigma_{\mathrm{ts}}$, 22.0 GPa, is rather high and of the same order of magnitude as the OUL value of 20 GPa reported for this material for the loading along the [111] direction [70]. A similar difference (12.7 GPa for the [001] direction and 27.3 GPa for the [111] one) was also found in Fe [71]. As we are not aware of any other calculations of strength for this material, the values shown in Table 1 are the first ab initio calculated values of strength for loading along the [001] direction also for the Fe${}_{3}$Al in the D0${}_{3}$ structure.

### 3.2. Mechanical Response of the Fe${}_{3}$Al/Fe-Al Nanocomposite

The next part of our ab initio simulations was focused to the determination of properties of the nanocomposite consisting of the ordered Fe${}_{3}$Al and the disordered Fe-Al phase. We applied the uniaxial deformation (OUD) and uniaxial loading (OUL) to the entire simulation cell shown in Figure 1 and compared the results with those summarized in the previous section for the individual phases. The stress-strain curves for the nanocomposite are depicted in Figure 2c and it can be seen that the shape of these curves is smooth for both OUD and OUL deformation models. Here, the uniaxial tensile strength $\sigma_{\mathrm{ts}}^{\mathrm{OUD}}$ = 23 GPa is almost the same as for the perfect disordered phases. This fact indicates that the strength found for uniaxial straining (here represented by triaxial loading state) of Fe${}_{3}$Al will be determined by the strength of the weaker disordered phase. On the other hand, the tensile strength for the uniaxial loading is $\sigma_{\mathrm{ts}}^{\mathrm{OUL}}$ = 5.6 GPa is located between the values of the strength calculated for Fe${}_{3}$Al and disordered Fe-Al phase. The maximum achieved strain values also indicate that the presence of the disordered structure increases the brittleness of the nanocomposite because these maximum strains are equal to those computed for this phase. In summary, if we neglect small increases of the strength in case of uniaxial loading we can conclude that the presence of the disordered phase has a negative effect on the mechanical characteristics, in particular a small reduction of the strength for uniaxial deformation and significant reduction of strains corresponding to the theoretical tensile strength.

### 3.3. Influence of Lateral Stresses on the Strength

The previous results revealed a very high influence of the lateral stresses $\sigma_{\mathrm{lt}}$ on the tensile strength $\sigma_{\mathrm{ts}}$ for all studied materials. Hence, in this section we analyze this effect in detail in order to clarify its impact on the mechanical characteristics of the studied Fe-Al-based systems. Here, using the quasi-static ( ab initio and molecular dynamics) simulations we performed several sets of the tensile tests where each individual test was realized under predefined constant value of the lateral stress $\sigma_{\mathrm{lt}}$. For example, to obtain the tensile strength for Fe${}_{3}$Al phase as a function of the lateral stress $\sigma_{\mathrm{lt}}$ we performed five tensile tests where each test was realized under a particular constant value of $\sigma_{\mathrm{lt}}$. As mentioned in Section 2 these values were chosen to be $\sigma_{\mathrm{lt}}$ = (0, 5, 10, 15, 20 GPa). We note that the tensile test obtain for $\sigma_{\mathrm{lt}}$ = 0 GPa is identical to the OUL model (the uniaxial loading).

**Figure 3:**
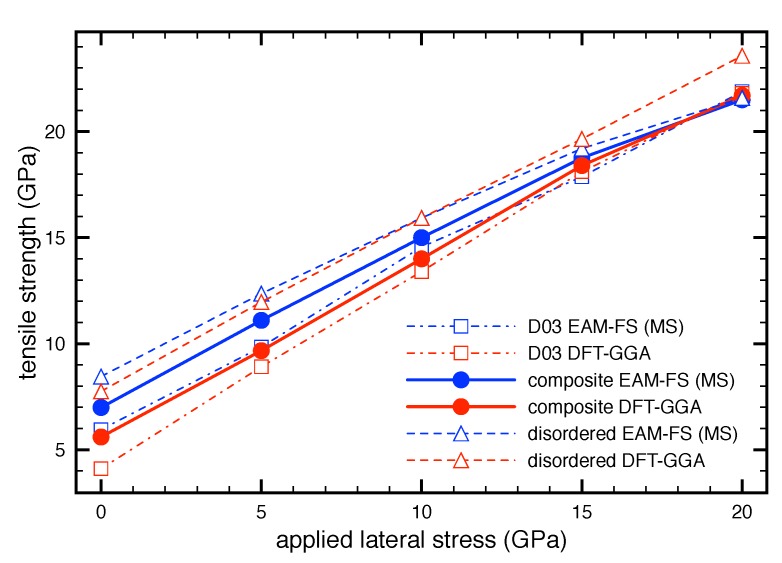
The effect of the transverse stresses on the tensile strength as studied by quantum-mechanical calculations (marked as DFT-GGA) and atomistic Embeded Atom Method (EAM) potentials (marked as EAM-FS).

The obtained tensile strength $\sigma_{\mathrm{ts}}$ as a function of the lateral stress $\sigma_{\mathrm{lt}}$ is illustrated in Figure 3 for Fe${}_{3}$Al, disordered Fe-Al phase as well as their nanocomposite. Here, the red curves represent the data obtained from the quantum-mechanical simulations whereas the blue ones are the data from the molecular quasi-static simulations. From these dependencies it is obvious that the tensile strength $\sigma_{\mathrm{ts}}$ for all structures linearly increases with increasing of the lateral stress $\sigma_{\mathrm{lt}}$ and therefore it can be approximated by linear functions $\sigma_{\mathrm{ts}}\left(\sigma_{\mathrm{lt}}\right)$ using the formula

(3) $$\sigma_{\mathrm{ts}}=\gamma \sigma_{\mathrm{lt}}+\sigma_{\mathrm{ts}}^{\mathrm{OUL}},$$

where the $\sigma_{\mathrm{lt}}$ is the selected value of the lateral stress, the γ represents the slope of this dependence and the $\sigma_{\mathrm{ts}}^{\mathrm{OUL}}$ is the tensile strength in corresponding direction for the OUL model. Let us note that the same equation was used by Černý and Pokluda for perfect bcc, fcc and hcp crystals [72,73]. Those papers also contain the values of the coefficients γ (denoted as $k_{\max}$ or *s* in the above mentioned papers) and the theoretical strengths for uniaxial loading $\sigma_{\mathrm{ts}}^{\mathrm{OUL}}$ (marked as $\sigma_{\max,0}$ or $\sigma_{r}$). Hence, the present results obtained for Fe-Al systems can be compared with perfect crystals, in particular with Fe. However, perfect Al crystal was not considered in Refs. [72,73]. For this reason, we supplement the present results with the data calculated for perfect fcc Al crystal, i.e., from the stress-strain curves for all deformation models considered in the present work.

The results obtained in Refs. [72,73] revealed that the most fcc and bcc crystals with linear dependencies of $\sigma_{\mathrm{ts}}$ on $\sigma_{\mathrm{lt}}$ have the slope γ mostly positive with higher values for bcc crystals than fcc ones. Interestingly, our results computed for perfect fcc Al crystal showed behavior similar to Ni or Cu where the tensile strength $\sigma_{\mathrm{ts}}$ is insensitive to the applied lateral stress $\sigma_{\mathrm{lt}}$. This means that the tensile strength $\sigma_{\mathrm{ts}}$ of Al always reaches the same value for all present deformation models. Of course, there are some small differences between the computed values, however, these differences are smaller than the stress convergence criteria introduced in the computational details, e.g., the $\sigma_{\mathrm{ts}}$ is always located in the range of 〈11.31;11.43〉 GPa. For this reason we consider the slope to be γ = 0. We must point out that the range of the lateral stress $\sigma_{\mathrm{lt}}$ used for Al is only within the values 0, 5, 10 GPa. On the other hand, the slope γ for perfect Fe is 0.63 [72] and more interestingly 0.79–0.89 for the Fe-Al-based alloys (Table 2). This means that the Fe-Al systems studied in this work are more sensitive to the lateral stress $\sigma_{\mathrm{lt}}$ than elemental Fe or Al. Thus, differences in loading conditions in Fe-Al systems lead to significant changes of the tensile strength $\sigma_{\mathrm{ts}}$ compared to Fe and Al.

The obtained results of the γ slope and the tensile strength $\sigma_{\mathrm{ts}}^{\mathrm{OUD}}$ are summarized in Table 2 together with the data for perfect Al and Fe crystals. Černý and Pokluda also proposed a simple formula for determination of the γ slope from the elastic constants [72].

(4) $$\gamma_{\mathrm{ec}}=\frac{\sigma_{\mathrm{iso}}-\sigma_{\mathrm{ts}}^{\mathrm{OUL}}}{\sigma_{\mathrm{iso}}}\approx 1-\frac{E_{100}}{3B}=\frac{2C_{12}}{C_{11}+C_{12}}$$

Using this equation we computed the values of γ for all studied systems and the results (marked as $\gamma_{\mathrm{ec}}$) are compared in Table 2 with those computed directly from the stress-strain dependencies. Because Equation (4) works only for cubic systems and the Fe-Al nanocomposite possesses orthorhombic symmetry we used average values of the elastic constants, i.e., $C_{11}$ = ($C_{11}$ + $C_{22}$ + $C_{33}$)/3, etc. (see the paper by Moakher and Norris [85]). As it can be seen, the slopes $\gamma_{\mathrm{ec}}$ agree very well with values of γ for the Fe-Al-based systems and perfect Fe crystal, however, the results do not agree for the perfect Al crystal. This difference can be easily explained via different crystal structure and the loading direction between Al and Fe-Al systems. Whereas the perfect Al crystal is subjected to the [001] deformation in fcc system then the present Fe-Al structures are deformed in [001] direction in bcc like system. Thus, both systems are completely different by means of the atomic ordering and the loading direction.

### 3.4. Temperature Effect on the Tensile Strength

**Figure 4:**
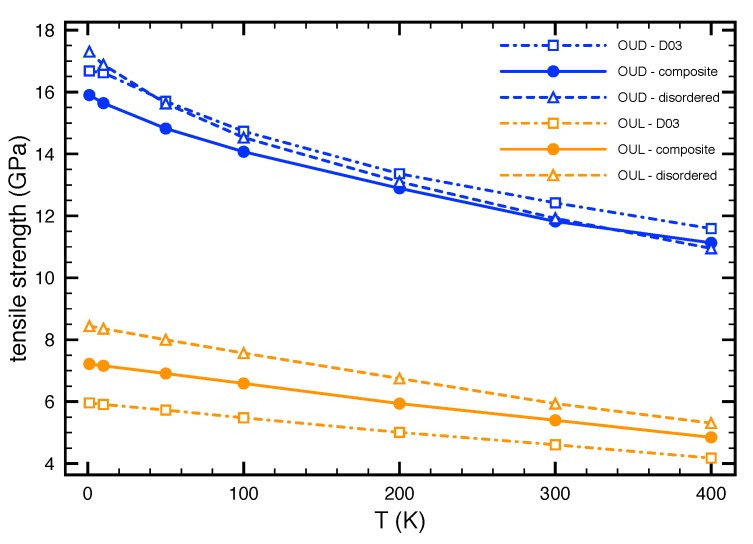
The tensile strength of Fe${}_{3}$Al and disordered Fe-Al phases together with their nanocomposite as functions of the temperature (the simulations were performed for 1, 10, 50, 100, 200, 300 and 400 K). Blue and yellow curves show the results obtained within OUD and OUL, respectively.

The previous results were computed under quasi-static simulations where temperature effect is neglected. To include temperature into our simulations we employed the molecular dynamics simulations. Using MD simulations we computed the tensile strength $\sigma_{\mathrm{ts}}$ for all studied materials as a function of temperature. The computed dependencies for OUD and OUL modes are depicted in Figure 4.

It can be seen that increasing temperature decreases the tensile strength for all studied phases and deformation models. As far as the Fe${}_{3}$Al and the OUD model are concerned, the strength decreases from 16.7 GPa (obtained from the *T* = 1 K simulations) to values approx. 12.5 GPa for temperature 300 K, i.e., only 75 % of the strength values obtained from the quasi-static simulations.

Similar effect can be observed for the uniaxial loading (OUL) where temperature also decreases the strength. According to these results it is obvious, that the quasi-static simulations for Fe${}_{3}$Al might overestimate the tensile strength when performed for a static lattice at T = 0 K and and the inclusion of the lattice vibrations at finite temperatures significantly decreases the tensile strength.

### 3.5. Elastic Constants

**Figure 5:**
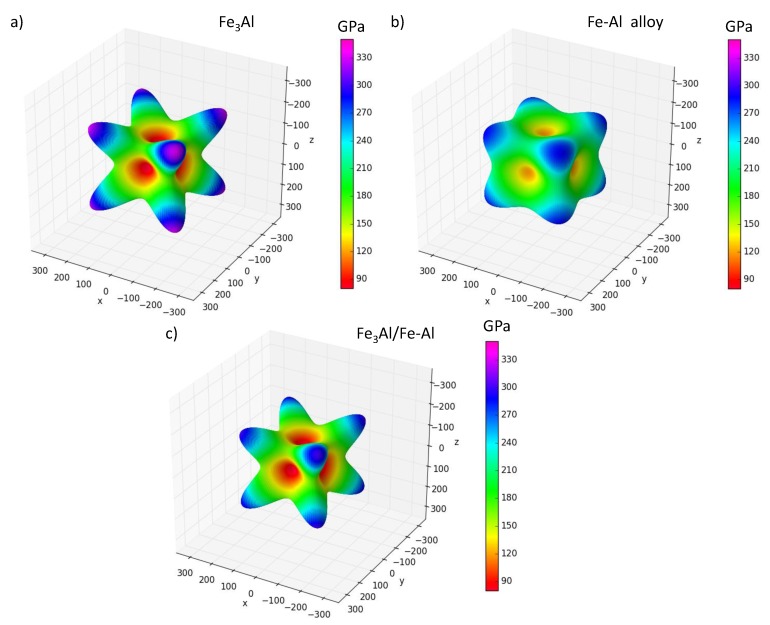
Directional dependencies of Young’s modulus of the ground-state configuration of Fe${}_{3}$Al compound (**a**); disordered Fe-Al (**b**) and the nanocomposite consisting of these two phases (**c**). All three dependencies were visualized using the SC-EMA software package (see it freely available at the web page scema.mpie.de) [88,89,90].

As was mentioned in the introduction, any crystal structure might become unstable even before reaching the maxima at stress-strain dependence. This instability might occur due to temperature effects (e.g., a phonon instability) or, specifically in the case of long-wave phonon modes close to the Γ-points, the elastic instability. The temperature effect was discussed in the previous section with the help of molecular dynamics simulations. However, the tensile strength might be also reduced due to elastic instability, i.e., failure to fulfill the stability conditions formulated using elastic constants $C_{ij}$. For this reason we computed $C_{ij}$ (using the stress-strain method [86]) as a function of the axial strain $\varepsilon_{ax}$ and if the conditions of elastic stability were not satisfied [87] the crystal structure was considered as unstable and the tensile strength was set equal to the corresponding value of stress. As far as the ground-state configurations of Fe${}_{3}$Al compound, the disordered Fe-Al and their nanocomposite are concerned, their elastic properties are summarized in Table 3 and visualized in the form of directional dependencies of Young’s modulus in Figure 5.

The calculated values for Fe${}_{3}$Al compound are $C_{11}$ = 211 GPa, $C_{12}$ = 161 GPa and $C_{44}$ = 139 GPa. The elastic constants computed for the disordered Fe-Al phase were projected onto a set of elastic constants possessing a cubic symmetry according to the rigorous mathematical theory by Moakher and Norris [85]. Similar concepts are often used in case of systems with any form of disorder (see e.g., Refs. [91,92,93,94,95]). The resulting cubic-symmetry elastic constants are $C_{11}$ = 217 GPa, $C_{12}$ = 131 GPa and $C_{44}$ = 120 GPa. As seen in Figure 5, both Fe${}_{3}$Al compound and Fe-Al nanocomposite have qualitatively the same type of the elastic anisotropy with the 〈111〉 directions being the hard ones while the 〈001〉 being the soft ones.

The nanocomposite consisting of these two phases turned out to have an orthorhombic symmetry and its elastic properties are characterized by elastic constants $C_{11}$ = 182 GPa, $C_{12}$ = 146 GPa, $C_{13}$ = 139 GPa, $C_{22}$ = 202 GPa, $C_{23}$ = 147 GPa, $C_{33}$ = 194 GPa, $C_{44}$ = 124 GPa, $C_{55}$ = 128 GPa and $C_{66}$ = 128 GPa. As it may be seen from Figure 5c, behavior of Young’s modulus of the nanocomposite is not very different from that of cubic symmetry. Therefore, we will use only three independent elastic constants for the nanocomposite in the text below.

Inspecting values for the ordered Fe${}_{3}$Al in Table 3 it is clear that there is rather significant scatter in the previously published results [96,97,98]. Moreover, all theoretical values differ quite significantly from the experimental data [99]. While also our predicted elastic constants differ from the experiment, they are nevertheless either the closest to the experimental values (in the case of $C_{11}$ and $C_{44}$) or nearly equal to the values which are the closest (as in the case of $C_{12}$). Now, it should be pointed out that both the theoretical and experimental elastic constants are only partly determined directly and, in fact, mostly indirectly. The theoretical values are, in the case of the stress-strain method, obtained from six sets of six coupled linear equations. On the other hand, the experimental data [99] listed in Table 3 were only indirectly derived from elastic characteristics $C^{\prime }$ = ($C_{11}$−$C_{12}$)/2, $C_{44}$ and $C_{L}$ = ($C_{11}$ + $C_{12}$ + 2$C_{44}$)/2 evaluated from direct measurements of the speed of sound. This aspect can introduce some systematic errors. Another reason for the differences from our values may consist in the use of a different computational software tool. Surprisingly, the best agreement is then reported in Ref. [100] where the authors combined the tight-binding linear-muffin-tin-orbital (TB-LMTO) method calculations and the inversion of the inter-atomic potentials based on the lattice inversion method. Despite of this agreement, their approach represents essentially only a pair-potential method what is seen in the fact that their Cauchy pressure $C_{12}$–$C_{44}$ is zero. On the other hand, let us note that from experimental elastic constants extrapolated to 0 K [99], a negative value of -7 GPa is obtained.

Using the anisotropic elastic parameters for each phase (see Table 3) we can also evaluate their homogenized isotropic polycrystal properties, such as bulk modulus *B*, Young’s modulus *E*, shear modulus *G* or Poisson’s ratio ν. These were calculated employing the SC-EMA software package (see it freely available at scema.mpie.de) [88,89,90] employing Green’s-function-based approach described, for example, in Ref. [89]. The SC-EMA software tool implements different homogenization techniques. First, two classical schemes of Voigt [101] (assuming equal strain in all grains) and Reuss [102] (supposing equal stress in all grains) are applied which represent the upper and lower bound, respectively. However, it also computes homogenized moduli for the cubic systems according to Hershey [103] scheme providing values which are typically close to the average of Voigt and Reuss ones (and often close to experiments in the case of texture-free samples). We used Hershey’s method for our analysis. Interestingly, the *B*, *G* and *E* parameters of the nanocomposite are not in between the values of the constituting phases. The values are either equal to the lower of the two values (such as bulk modulus *B*) or even lower (*G* and *E*) than the lowest values predicted for individual phases. The interfaces thus represent elastically weaker links within the composite.

Comparing our values of polycrystalline elastic moduli with the previously published ones we observe that they are quite different. In particular, B/G ratio from Ref. [96] is very low but this is the consequence of the use of Voigt homogenization scheme which provides the upper limit of the shear modulus *G* while the bulk modulus *B* is equal to the same value for Voigt, Reuss and Hershey method in the case of cubic-symmetry systems (see them compared, e.g., in our paper [104]).

The values of Poisson’s ratio ν, the B/G ratio as well as the values of the Cauchy pressure $C_{12}$–$C_{44}$ can be further used to estimate ductile/brittle behavior of the studied materials. In particular, the B/G ratio was introduced by Pugh [105] based on empirical data and the value over 1.75 was suggested to indicate a ductile behavior while lower values mean a brittle type. Cauchy pressure [106] can also be indicative regarding the ductility of materials (with positive values associated with ductile behavior). Ductility is also associated with larger values of Poisson’s ratio, typically over 0.31–0.32. Two of these three parameters, B/G and the Cauchy pressure, indicate that all three studied materials are ductile (B/G> 1.75, $C_{12}$–$C_{44}$ positive) but the value of Poisson’s ratio is only 0.285 for the disordered Fe-Al phase and the values for ordered Fe${}_{3}$Al and the nanocomposite are within the border range of values (0.31–0.32). Again, let us note that the experimental value of the Cauchy pressure obtained from measurements of elastic constants extrapolated to 0 K is slightly negative (−7 GPa) and becomes negative up to 300 K (−1.1 GPa [99]).

Now, as far as the loaded states are concerned, the first stability condition is not satisfied [87] in Fe${}_{3}$Al compound when the axial strain ε reaches 0.26 and the phase is thus mechanically unstable. The instability is related to the $C^{\prime }$ elastic constant. There is no doubt that the tensile strength $\sigma_{\mathrm{ts}}$ determined from the stress-strain curves for Fe${}_{3}$Al in Figure 2a must be adjusted (decreased) according to this instability. In the corresponding stress-strain curve the axial strain value $\varepsilon_{\mathrm{ax}}$ = 0.26 corresponds to 22 GPa and hence this value is the reduced tensile strength $\sigma_{\mathrm{ts}}$ for the quasi-static simulations (0 K temperature). The corresponding value is also highlighted in Figure 2a and marked as the “instability”. This adjustment of the tensile strength $\sigma_{\mathrm{ts}}$ clearly demonstrates that the stress-strain curves obtained from the quasi-static simulations are not sufficient to determine the strength and other possible instability effects should be included.

Regarding the disordered phase and the nanocomposite, the instabilities occur in the case of the former for the OUD conditions and in the case of the latter under both types of simulated conditions (OUD and OUL). The instability of the nanocomposite loaded under OUD conditions is related to both $C^{\prime }$ and $C_{66}$ while the other two instabilities are related solely to $C^{\prime }$.

### 3.6. Fracture of the Nanocomposite upon Loading

The last part of our study is devoted to identification of the weakest bonding in the nanocomposite, i.e., the location where a fracture appears and two new surfaces are created. This place is characterized as a plane with the lowest cleavage stress. The selected deformation modes use the optimization of ionic positions in the simulation cell during each strain increment and hence it is not necessary to predict its location as in the models where the ionic optimization is not employed. In deformation modes used in the present work the cleavage plane is found as a result of optimization of ionic positions when the critical strain is reached. Thus, the locations of the cleavage plane in the nanocomposite and the disordered phases were found via a detail examination of their configurations after each strain increment.

However, before describing the cleavage plane location we would like to point out that the stress-strain curves obtained from the atomistic simulations can be separated into two groups according to character of their dependencies. The first one is represented by smooth and continual increases of the stress up to its maximum followed by smooth decreases. Thus, the stress-strain curve does not contain any sudden drops or increases and its entire shape is smooth. This behavior is typical for tensile tests in atomistic simulations of perfect crystals where no irregularities such as vacancies, interfaces or grain boundaries, are present in the structure. As a typical example we may mention the stress-strain curve for the perfect Fe${}_{3}$Al compound in Figure 2a for both OUD and OUL deformation modes. This material behavior is often found also in structures with defects subjected to uniaxial loading only. This type of the dependence usually indicates that the studied material has many degrees of freedom and instead of a brittle fracture it rather transforms into a different structural configuration [65]. This is also the case of the stress response of the nanocomposite in Figure 2c for the OUL mode. On the other hand, the second type of the behavior is represented by stress-strain curves that contain abrupt drops of the stress immediately after its maximum. This drop represents a structure failure which usually means that the bonds between atoms at the weakest plane were broken and two new surfaces were created, i.e., a fracture appears. This behavior is typical for crystal structures containing defects that are subjected to triaxial loading state.

**Figure 6:**
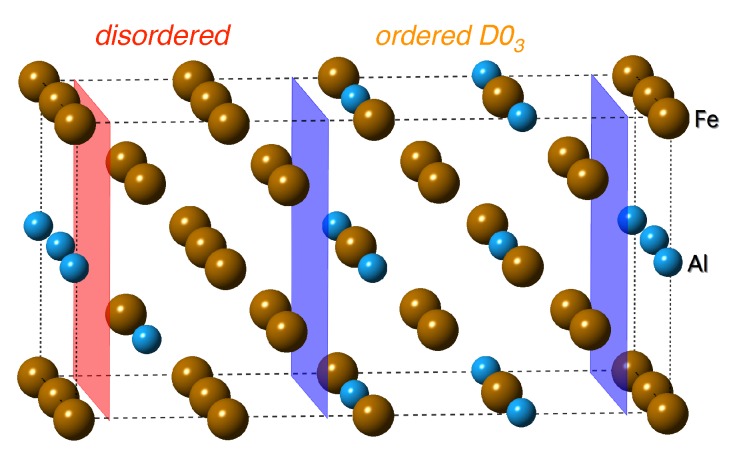
The location of the cleavage plane highlighted by the red plane. The blue planes mark the interfaces between D0${}_{3}$ and disordered phases.

The detailed examination of the stress-strain curves displayed in Figure 2a and of corresponding structure configurations reveals that Fe${}_{3}$Al phase does not exhibit any abrupt drop and therefore there is no fracture around the maximum of the stress. On the other hand, the nanocomposite subjected to uniaxial deformation (OUD) fails due to fracture (there is an abrupt drop in Figure 2c). Fracture is located at the cleavage plane with the lowest cleavage stress. This conclusion was confirmed when examining the structure at each strain increment and it has been found that the fracture appears in the disordered phase as it is marked in Figure 6. The location of the plane with the lowest cleavage stress is highlighted by the red color. Therefore, the disordered phase represents the weakest part of the studied nanocomposite in terms of tensile loading. The cleavage plane is identical to that one obtained for the perfect disordered phase. It is also evident that there is no fracture for uniaxial loading.

## 4. Conclusions

We have performed an ab initio study of ordered Fe${}_{3}$Al compound with the D0${}_{3}$ structure, a disordered Fe-Al solid solution with 18.75 at.% Al and of a nanocomposite consisting of an equal molar amount of both phases under uniaxial loading conditions along the [001] direction. By comparing the behavior of individual phases with that of the nanocomposite we find that the disordered Fe-Al phase represents the weakest point of the studied nanocomposite in terms of tensile loading. The cleavage plane of the whole nanocomposite is identical to that identified when loading solely the disordered Fe-Al phase.

It also turns out that the strength of Fe${}_{3}$Al compound strongly depends on triaxiality of the loading state, i.e., increasing of the lateral stresses significantly increases the tensile strength. This dependence has a linear character and therefore it can be described via a simple formula. For Fe-Al-based materials studied here, its slope is higher than for that perfect Fe crystal in the corresponding direction and completely different when compared with perfect Al crystal. Therefore, the strain response of the Fe-Al alloy and of a nanocomposite cannot be predicted on the basis of the knowledge of the strain response of Fe or Al.

The mechanical stability is found to be closely interlinked with elastic constants (in particular $C^{\prime }$ and/or $C_{66}$) which soften with increasing uniaxial loading and eventually violate stability conditions. Next, we also conclude that there is no brittle-type fracture for uniaxial loading and the nanocomposite transforms rather continuously and diffusionlessly into a face-centered cubic-like structure, although it fails due to fracture under uniaxial deformation. Finally, our atomistic Embedded Atom Method (EAM) simulations show that temperature significantly affects the mechanical properties compared to those obtained from quasi-static simulations. For example, at room temperature of 300 K the strength decreases to as low as 75% of the zero-Kelvin static lattice value.

## Figures and Tables

**Table 1:** The tensile strengths $\sigma_{\mathrm{ts}}$ in the [001] direction for the Fe${}_{3}$Al compound and the disordered Fe-Al phase with 18.75 at.% Al together with their nanocomposite from ab initio calculations, from quasi-static simulations and from the molecular dynamics simulations at temperature of 1 K. The table contains the tensile strengths obtained from maximum at stress-strain dependence (ab initio; except of the value of 24.7 GPa for Fe${}_{3}$Al OUD, which corresponds rather to a structural transformation), the ab initio tensile strength obtained from elastic instability (ab initio + ei), molecular static (MD (qs)) and molecular dynamics at the temperature of 1 K.

	ab initio	ab initio + ei	MD (qs)	MD (1 K)
Fe${}_{3}$Al OUD	24.7	22.0	18.6	16.7
Fe${}_{3}$Al OUL	4.1	4.1	6.0	6.0
Fe-Al disordered OUD	22.9	-	17.7	17.3
Fe-Al disordered OUL	7.8	-	8.5	8.4
nanocomposite OUD	23.0	21.4	16.6	16.0
nanocomposite OUL	5.6	5.5	7.0	7.2

**Table 2:** The coefficients γ and $\gamma_{\mathrm{ec}}$ from Equation (3) that were determined directly from the stress-strain dependence and via Equation (4), respectively. The Table also contains the tensile strengths $\sigma_{\mathrm{ts}}^{\mathrm{OUL}}$ for elemental Fe (bcc) [71,72,74,75,76,77,78], Al (fcc) (present calculations and Refs. [79,80,81,82,83]) and Fe-Al-based systems. Most values of strength given in the Table correspond to the maximum of stress at the stress-strain curve. All values without a reference come from the present work. The $\gamma_{\mathrm{ec}}$ for Al was computed from Equation (4) and the elastic constants were taken from Ref. [84] ($C_{11}$ = 123 GPa, $C_{12}$ = 70.8 GPa and $C_{44}$ = 30.9 GPa). The values of $\gamma_{\mathrm{ec}}$ for Fe-Al systems were obtained from the elastic constants calculated here (see Table 3).

	γ	$\gamma_{\mathrm{ec}}$	$\sigma_{\mathrm{ts}}^{\mathrm{OUL}}\left(\mathrm{GPa}\right)$
elemental Fe (bcc)	0.63 [72]	0.67 [72]	12.7 [71,74,77], 14.2 [75], 12.6 [76], 12.4 [72,78]
elemental Al (fcc)	0.00	0.73	12.6 [79], 12.1* [79], 12.92 [80]
			9.20** [80], 11.6 [81], 9.0* [82], 11.33 [83], 11.4
Fe-Al - nanocomposite	0.82	0.86	5.5
Fe${}_{3}$Al - D0${}_{3}$	0.89	0.87	4.1
Fe-Al - disordered	0.79	0.75	7.8

* corresponding to elastic instabilities occurring prior to reaching the maximum stress at the stress-strain curve, ** obtained from a phonon instability with finite wave vector.

**Table 3:** The computed anisotropic elastic constants $C_{11}$, $C_{12}$ and $C_{44}$ together with homogenized bulk modulus *B*, Young’s modulus *E*, shear modulus *G*, Poisson ratio ν and the Cauchy pressure $C_{12}$–$C_{44}$. The Table also contains previous theoretical results [96,97,98] as well as available experimental data [99]. All elastic constants (except for Poisson’s ratio) and moduli are given in GPa.

	$C_{11}$	$C_{12}$	$C_{44}$	*B*	*G*
ordered Fe${}_{3}$Al-D0${}_{3}$	211	161	139	178	73
	225 [96]	160 [96]	147 [96]	180 [96]	101 [96]
	283 [97]	206 [97]	149 [97]	232 [97]	87 [97]
	285 [98]	208 [98]	151 [98]	233 [98]	88 [98]
	159 [100]	138 [100]	138 [100]	144* [100], 170** [100]	–
experiment	179 [99]	131 [99]	138 [99]	147 [99]	–
disordered Fe-Al	217	131	120	160	80
nanocomposite	193	144	127	160	68
	*E*	ν	*B*/*G*	$C_{12}$–$C_{44}$	
ordered Fe${}_{3}$Al-D0${}_{3}$	193	0.319	2.438	22	
	347 [96]	0.179 [96]	1.782 [96]	13 [96]	
	–	0.400 [97]	2.632 [97]	57 [97]	
	234 [98]	0.333 [98]	2.65 [98]	57 [98]	
disordered Fe-Al	206	0.285	2.000	11	
nanocomposite	178	0.314	2.353	17	

* derived from elastic constants, ** directly from TB-LMTO calculations.
